# *n*–3 Polyunsaturated Fatty Acid Amides: New Avenues in the Prevention and Treatment of Breast Cancer

**DOI:** 10.3390/ijms21072279

**Published:** 2020-03-26

**Authors:** Cinzia Giordano, Pierluigi Plastina, Ines Barone, Stefania Catalano, Daniela Bonofiglio

**Affiliations:** 1 Department of Pharmacy, Health and Nutritional Sciences, 87036 Arcavacata di Rende, Italy; cinzia.giordano@unical.it (C.G.); pierluigi.plastina@unical.it (P.P.); ines.barone@unical.it (I.B.); stefcatalano@libero.it (S.C.); 2 Centro Sanitario, University of Calabria, 87036 Arcavacata di Rende, Italy

**Keywords:** omega−3 polyunsaturated fatty acids, omega−3 polyunsaturated fatty acid derivatives, omega−3 polyunsaturated fatty acid conjugates, omega−3 polyunsaturated fatty acid amides, breast cancer, cannabinoid receptors, peroxisome proliferator-activated receptor gamma

## Abstract

Over the last decades a renewed interest in *n*−3 very long polyunsaturated fatty acids (PUFAs), derived mainly from fish oils in the human diet, has been observed because of their potential effects against cancer diseases, including breast carcinoma. These *n*−3 PUFAs mainly consist of eicosapentaenoic acid (EPA) and docosahexaenoic acid (DHA) that, alone or in combination with anticancer agents, induce cell cycle arrest, autophagy, apoptosis, and tumor growth inhibition. A large number of molecular targets of *n*−3 PUFAs have been identified and multiple mechanisms appear to underlie their antineoplastic activities. Evidence exists that EPA and DHA also elicit anticancer effects by the conversion to their corresponding ethanolamide derivatives in cancer cells, by binding and activation of different receptors and distinct signaling pathways. Other conjugates with serotonin or dopamine have been found to exert anti-inflammatory activities in breast tumor microenvironment, indicating the importance of these compounds as modulators of tumor epithelial/stroma interplay. The objective of this review is to provide a general overview and an update of the current *n*−3 PUFA derivative research and to highlight intriguing aspects of the potential therapeutic benefits of these low-toxicity compounds in breast cancer treatment and care.

## 1. Introduction

*n*−3 fatty acids are a group of polyunsaturated fatty acids (PUFAs) that play crucial roles in cell membrane structure, fluidity, and cell signaling. The designation *n−*3 is structural, referring to the position of the closest double bond with respect to the methyl group. The most abundant dietary *n*−3 PUFAs are alpha linolenic acid (ALA) (18:3*n*−3), whereas the longer chain *n*−3 PUFAs eicosapentaenoic acid (EPA; 20:5*n*−3) and docosahexaenoic acid (DHA; 22:6*n*−3), commonly referred to as marine fatty acids, are most efficiently obtained from fatty cold water fish such as salmon and mackerel. Unless EPA and DHA are directly ingested, they may be derived from ALA through the activity of desaturases and elongases that have a greater affinity for ALA than for *n*−6 linoleic acid (LA, 18:2*n*−6). However, since intake of LA is commonly 10-fold higher, due to the Westernized dietary habits, more *n*−6 arachidonic acid (AA) than EPA and DHA is formed. High amounts of LA are present in vegetable oils, seeds, and nuts, whereas ALA is especially contained in leafy vegetables, seeds, walnuts, soybeans, flaxseed and seed oils. Dietary intake of *n*−3 PUFAs has been shown to have health benefits against common inflammatory diseases and to inversely correlate with the overall risk of different types of malignancies such as colorectal, prostate, and breast cancer in human studies [1,2,3,4,5,6]. Particularly, EPA and DHA have been investigated as potential dietary-based agents for breast cancer prevention [7], and they have been shown to exhibit multiple anticancer mechanisms of action, including inhibition of cell proliferation [8], metastasis [8,9], blood vessel formation [10], inflammation [11], as well as induction of cell cycle arrest and apoptosis [8,12,13]. Previous data have been reported that DHA inhibits mammary tumor formation in a mouse polyoma virus middle T antigen induced mammary cancer mouse model [14], reduces formation of mammospheres, and induces apoptosis of tumor-initiating cells (TICs) in human triple negative breast cancers [15]. However, it has already been shown that consumption of *n*–3 PUFAs also stimulates the formation of specific *n*–3 PUFA conjugates with ethanolamine, dopamine, serotonin, or other amines. In most cases, information is limited to a demonstration of their existence and, even though basic molecular properties have not been fully elucidated yet, these mediators may provide promising new leads to the field of inflammatory and cancer diseases. From their structural analogy to the corresponding arachidonic acid *N*-conjugates, it is likely that there is a number of candidate receptors to which *n*–3 amides may show affinity, including cannabinoid receptors (CBs), G coupled protein receptors (GPRs), transient receptors potential channel type V1 (TRVP1), and peroxisome proliferator activated receptors (PPARs) [16,17,18]. The ethanolamine and dopamine conjugates of EPA and DHA have been the most investigated thus far [17,19,20,21,22,23]. Evidence is accumulating that DHA-ethanolamine (*N*-docosahexaenoylethanolamine, DHEA), DHA-dopamine (*N*-docosahexaenoyldopamine, DHADA), DHA-serotonin (*N*-docosahexaenoylserotonin, DHA−5HT), and other *n*–3 PUFA amides merit further studies in relation to their properties as anti-inflammatory and anticancer agents. In the present study, we systematically reviewed the biological effects of some conjugates of EPA and DHA with particular focus on their potential anti-inflammatory and antineoplastic properties within the breast tumor microenvironment highlighting their potential as anticancer agents from a pharmacological or nutritional perspective.

## 2. Search Strategy and Data Extraction

**Figure 1:**
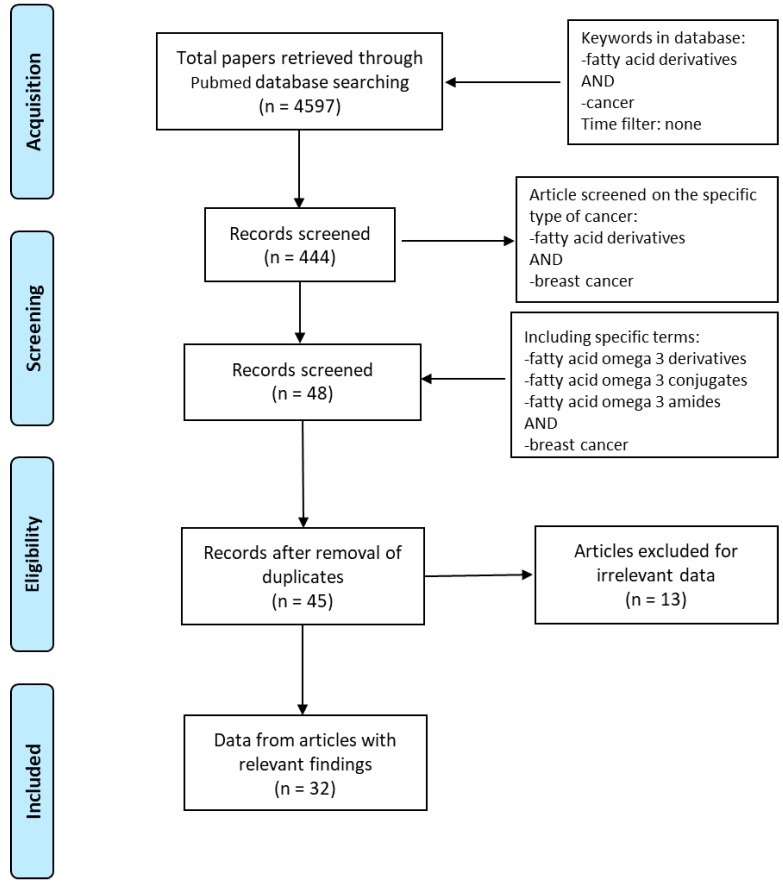
Flow diagram of the search strategy. Keywords used in PubMed database were “Fatty acid derivatives” AND “cancer”, “Fatty acid derivatives” AND “breast cancer”, “Fatty acid omega 3 derivatives” OR “Fatty acid omega 3 conjugates” OR “Fatty acid omega 3 amides” AND “breast cancer”. Finally, 32 papers were deemed eligible to be reviewed.

Based on the Preferred Reporting Items for Systematic Reviews and Meta-Analyses (PRISMA) guidelines, we explored PubMed database in order to access the relevant data between 1997 and 2020. The literature search was performed using the following keywords: “fatty acid derivatives” AND “cancer” obtaining 4597 papers. We identified full-text articles written in English. A total of 444 published papers were restricted when we used as keywords “fatty acid derivatives” AND “breast cancer”. In order to focus our attention on eicosapentaenoic and docosahexaenoic acids, “fatty acid omega 3 derivatives” AND “breast cancer” or “fatty acid omega 3 conjugates” AND “breast cancer” or “fatty acid omega 3 amides” AND “breast cancer” were used. After removing the duplicates, the articles were screened based on their relevance to the topic and all irrelevant papers were excluded. All discrepancies were subjected to discussion until proper conclusions were made in each case. A final number of 32 articles met all the inclusion criteria and were found suitable to be reviewed (Figure 1). Data extraction was performed, and the key findings related to the ‘in vitro’ and ‘in vivo’ studies from original papers showing the antineoplastic effects of n−3 PUFA derivatives in breast cancer are presented in Table 1. The data reviewed were organized in separate sections including (1) formation and turnover of *n*–3 PUFA amides; (2) biological activities and potential targets of *n*–3 PUFA amides; (3) molecular mechanisms of selective action of EPA and DHA conjugates on epithelial breast cancer cells and tumor microenvironment. Finally, because of the beneficial health effects of *n*–3 PUFA amides, we highlighted their potential as breast cancer preventive and/or therapeutic agents.

### 2.1. Formation and Turnover of n–3 PUFA Amides

Over the last years, *N*-acyl amines of DHA and EPA and other *n*−3 PUFA derived conjugates with ethanolamine, serotonin, alanine, serine, histidine, GABA, glutamic acid, or dopamine have attracted much attention because of their potential roles in several pathophysiological conditions, suggesting that they could represent a new additional class of endogenous signaling molecules [24,37]. These compounds, that are amphiphilic molecules, characterized by a polar head group and a hydrocarbon tail, can be derived from the conversion of their corresponding *n*−3 PUFAs. There are different enzymes involved in endogenous biosynthesis and metabolism of *n*–3 PUFA amides, such as phospholipases and hydrolases, but also specifically the fatty acid amide hydrolase (FAAH), although this reversed hydrolysis would likely occur at significantly higher concentrations. Indeed, since the incorporation of fatty acids into membranes can be modulated by their proportional abundance in the diet, a link with dietary intake does exist. Consistently, dietary intake of *n*−3 PUFAs greatly enhances the production of their corresponding ethanolamides as demonstrated in the liver and jejunum of rats fed high fish oil diet [38] and in the brain of newborn piglets after feeding milk formula rich in EPA and DHA [39]. In addition, the formation of *N*-acylethanolamines from their precursor fatty acids has been reported in human adipocytes [40], in hippocampal neuron cultures [41,42], as well as in human breast and prostate cancer cells [29]. It has been reported that several fatty amides can be rapidly synthesized from their fatty acid precursors into membranes, released on demand, and quickly broken down [17,43], suggesting that the local availability of both specific types of enzymes and fatty acid precursors may determine their product formation and hence bioactivity. In clinical studies in healthy volunteers with daily intake of fish oil food supplements (480 mg EPA plus 360 mg DHA per day for 3 weeks) doubled plasma DHEA levels have been found [37]. However, further studies are needed to confirm which type of biosynthesis drives their formation predominantly in vivo.

The *n*−3 PUFA derivatives are further metabolized via non-enzymatic and enzymatic pathways. Non-enzymatic generation of electrophilic derivatives of long chain PUFAs occurs through free-radical-catalyzed lipid peroxidation of both free and esterified fatty acids, while the primary degrading enzyme is FAAH, which is localized on the endoplasmatic reticulum [44] or located on cytoplasmic lipid droplets [44,45]. Next to hydrolysis, the breakdown of *n*–3 PUFA amides follows different enzymatic mechanisms leading to the formation of oxygenated derivative compounds. Specifically, three enzyme families are mainly responsible for the oxygenation of *n*−3 PUFAs, namely, cyclooxygenases (COX), lipoxygenases, and cytochromes P450 [46,47,48]. Studies have extensively investigated the generation of oxidized metabolites that have been produced through these enzymes [49,50]. Endogenous generation of electrophilic oxo-derivatives of *n*−3 PUFAs has been observed in in vitro and ex vivo human models and dietary supplementation of *n*−3 PUFAs has been also reported to increase their formation [50]. Interestingly, such *n*−3 PUFA derivatives act as mediators of the beneficial actions observed upon dietary administration of *n*−3 PUFAs and, therefore, there has been a growing interest also in characterizing their formation and signaling actions in health and diseases.

**Figure 2:**
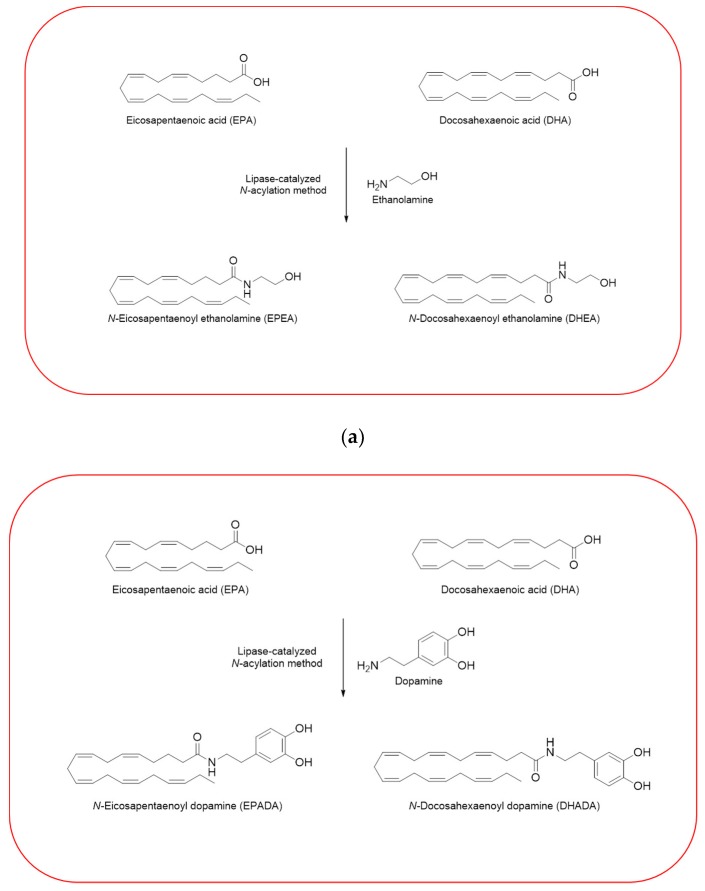
Synthesis of *n*–3 polyunsaturated fatty acid (PUFA) amides. Chemical structures of two pairs of *n*–3 PUFA amides with ethanolamine and dopamine, *N*-acylethanolamines (*N*-docosahexaenoylethanolamine (DHEA) and *N*-eicosapentaenoylethanolamine (EPEA)) (**a**) and *N*-acyldopamines (*N*-docosahexaenoyldopamine (DHADA) and *N*-eicosapentaenoyldopamine (EPADA)) (**b**).

Given the importance to unravel the mechanisms of action of *n*−3 PUFA derivatives, different related compounds were synthesized by several researches over the last decades [26,27,28,30,33]. In line with the growing interests in these intriguing molecules, we have also synthesized and then tested some of them in different cell line models. Using a previously described enzymatic procedure [51], we have obtained two pairs of *n*–3 PUFA amides with ethanolamine and dopamine, *N*-acylethanolamines (DHEA and EPEA) and *N*-acyldopamines (DHADA and EPADA), respectively (Figure 2).

Both DHEA and EPEA were prepared from ethanolamine and their corresponding DHA and EPA, respectively. Briefly, the method is based on a direct condensation reaction between ethanolamine and the fatty acid (molar ratio 1:1), carried out at 40 °C in hexane, for 15 h, using immobilized Candida Antarctica Lipase B (CALB) as the catalyst. Although the synthesis of *N*-acyldopamines has been firstly reported by Bezuglov et al. [52], we have modified our previously reported lipase-catalyzed *N*-acylation method [51] to attain DHADA and EPADA for our experimental purposes. Briefly, dopamine hydrochloride, triethylamine and the appropriate fatty acid (molar ratio 1:1.5:1) were incubated at 50 °C in 2-methyl-2-butanol as solvent for 48 h, using CALB as the catalyst. After evaporating the solvent under reduced pressure, the products were purified by column chromatography on silica gel and verified by ESI-MS and NMR for their authenticity. We reviewed in the following sections the biological activities and potential targets of *n*−3 derivatives from DHA and EPA in inflammation and breast cancer. Molecular mechanisms of selective action of EPA and DHA conjugates on epithelial breast cancer cells and tumor microenvironment were also discussed.

### 2.2. Biological Activities and Potential Targets of n–3 PUFA-Derived Fatty Amides

Increasing evidence suggests that many *n*–3 PUFA amides are important signaling molecules in animals and humans, mediating a wide range of biological effects [53]. Based on the ubiquitous presence of the enzyme systems that are able to synthesize amides, the hypothesis has been raised that any available long-chain fatty acid can be amidated with any amino acid or related compounds. However, in vivo fatty acid amides are known to occur in fluctuating mixtures of structurally related molecules with pleiotropic and tissue specific activities. Indeed, the number of amides detected in tissues is rapidly increasing and several *n*−3 PUFA conjugates with amino acids and neurotransmitters have been found in mammal tissues, in plasma, and recently also in human milk [37,53]. For many of these molecules, relatively little is known about their biological significance and in many cases, only in vitro data are available, often obtained from testing single compounds. Regarding the fatty acid moiety, the majority of studies have focused on conjugates of DHA and EPA with ethanolamine or dopamine but conjugates of DHA with serotonin have also received attention in this review.

#### 2.2.1. *N*-Acylethanolamines, DHEA and EPEA

Several fatty acid amides show anti-inflammatory or general immune modulating properties [43,54,55]. Data from in vitro studies demonstrated the ability of DHEA and EPEA to inhibit nitric oxide (NO) release from stimulated RAW264.7 macrophages. Interestingly, DHEA was the most potent among a series of compounds tested and showed a concentration-dependent inhibition of NO release, while DHA showed a much smaller effect. DHEA can also inhibit the release of monocyte chemoattractant protein-1 (MCP-1) from lipopolysaccharide (LPS)-activated mouse peritoneal macrophages. These effects were, at least in part, mediated at a transcriptional level, since iNOS and MCP-1 mRNA levels were found to be reduced [56]. In addition, the spectrum of anti-inflammatory activities of DHEA included the modulation of interleukin-6 (IL-6) and eicosanoid release in macrophages, indicating that the anti-inflammatory action of this compound might be mediated through involvement of different pathways [57]. It has been previously reported that DHA and EPA can be converted by differentiated 3T3-L1 adipocytes to their corresponding *N*-acyl ethanolamines DHEA and EPEA, respectively, which are able to reduce LPS-induced production of MCP-1 and IL-6 [40]. Interestingly, DHEA and EPEA tissue levels were found to increase after an inflammatory stimulus in mice fed fish oil [58], suggesting that these compounds may have a role as endogenous anti-inflammatory mediators.

#### 2.2.2. *N*-Acyldopamines, DHADA and EPADA

Similar to DHEA, DHADA is also of interest due to its potential physiological properties that are linked to inflammation in the nervous system [59]. Dang and coworkers demonstrated that compounds bearing a dopamine head group were the most active in reducing pro-inflammatory markers among a series of structural analogues [60]. In rodent macrophages and microglia cells, DHADA suppressed in a concentration-dependent manner the protein levels of pro-inflammatory markers, such as NO, MCP-1, macrophage-inflammatory protein 3α (MIP3A or CCL20), IL-6 as well as levels of prostaglandin E2 (PGE2), which is a cyclooxygenase-2 (COX-2) product that promotes inflammation [61]. These findings suggest that DHA and dopamine, the most abundant fatty acid and an important neurotransmitter in the brain, could combine in a lipid mediator conjugate able to modulate neuro-inflammation. Recently, Augimeri and coworkers have added EPDA as a member of the group of immune-modulating *n*-3 PUFA derived compounds for its anti-inflammatory properties exerted in mouse RAW264.7 and human THP-1 macrophages stimulated with LPS. EPDA was found to suppress the production of MCP-1, IL-6, NO and CCL20 in RAW264.7 cells, demonstrating a possible involvement of COX-2-mediated processes in the apparent anti-inflammatory pathways. Moreover, the biological effects observed in the murine macrophages was also reproduced in a cell line of human origin, demonstrating that MCP-1 and IL-6 production was decreased upon treatment with EPDA. These inhibitory activities were regulated at a transcriptional level in macrophages, as a significant reduction in mRNA expression levels of genes investigated upon EPDA treatment was found [62]. These data underline for the first time that EPDA possesses immune-modulating properties in human macrophages.

#### 2.2.3. *N*-Acylserotonin, DHA-5HT

Another conjugate of DHA that has been recently included in the class of immune-modulating mediators is DHA-5HT. This molecule has been firstly detected as an endogenous compound in mice and pig intestine especially in the jejunum and ileum at increasing concentrations after receiving *n*-3 PUFA enriched diet [63]. Biological activities and potential targets of DHA-5HT were studied in mouse model of LPS-stimulated macrophages, in which DHA-5HT significantly attenuated production of IL-6, PGE2, interleukin-1beta (IL-1β), and interleukin-23 (IL-23), important mediators in triggering the development and maintenance of pathogenic T helper 17 (Th17) cells [64]. Notably, these effects were translated in human peripheral blood mononuclear cells in which this conjugate had the ability to inhibit the release of interleukin-17 (IL-17) and CCL20 [65]. Since Th17 cells have important pathophysiological functions, including protection against pathogens during chronic inflammatory diseases, these findings suggest that DHA-5-HT might have a role in the regulation of immune processes such as in inflammatory intestinal diseases. Nowadays, evidence supports the anti-inflammatory properties of *n*-3 fatty acid conjugates on macrophages, mainly due to the reduced secretion of the most important pro-inflammatory mediators, thus modulating M1 macrophage polarization induced by LPS. It is well known that LPS-induced inflammation is triggered by the direct contact between LPS and its receptor toll-like receptor 4, leading to the activation of downstream signaling cascades regulating the expression of cytokines. However, inflammation is a complex process in which different receptors, signaling pathways, and transcription factors are involved. Indeed, *n*–3 PUFA amides possess some affinity to CBs and non-CB receptors, such as GPRs, TRVP1, and PPARs [66,67,68]. Interestingly, among PPARs that are members of the nuclear receptor superfamily and function as ligand-activated transcription factors [69], PPARγ is a subset of the PPAR family, mainly expressed in adipose tissue, mammary gland, colon, and the immune system [70,71]. PPARγ binding to ligands including naturally occurring compounds such as PUFAs and eicosanoids, as well as synthetic activators, regulates the expression of target genes by binding to DNA sequence elements, termed PPAR response elements (PPREs) [72]. PPREs have been identified in the regulatory regions of a variety of genes that are involved in lipid metabolism and homeostasis, but also in inflammatory responses [69,73]. Evidence supports that *n*−3 PUFAs have been found to promote macrophages M2 polarization through PPARγ activation [74]. In addition, *n*–3 PUFA amides reduce the expression of pro-inflammatory markers partially through PPARγ activation [75]. However, more research is needed to describe the mechanisms of action of these molecules in depth.

It has been largely reported that inflammation, recognized as a hallmark of cancer, is able to predispose tumor development and progression by well-orchestrated reciprocal interactions among cancer cells and surrounding stromal inflammatory cells within the tumor microenvironment. Several lines of evidence indicate that DHA and EPA, apart from their anti-inflammatory actions, have beneficial effects in cancer [76,77,78,79,80,81,82], suggesting their potential role either in cancer prevention or therapy. The main anti-neoplastic activities of *n*−3 PUFAs include the alteration of the composition of cell surface membrane and lipid raft disruption, the modulation of COX activity, the increased cellular oxidative stress, and the binding and activation of different receptors, including CBs, GPRs, TRVP1, and PPARs [83]. However, EPA and DHA can also elicit anticancer effects by the conversion in situ to their corresponding derivatives. For instance, it has been demonstrated in cancer models that DHA and EPA can be converted into their respective ethanolamides [29]. We will extensively examine in the following section the studies that have investigated the role of conjugates of PUFAs with ethanolamine, mono-amine neurotransmitters, or amino acids in influencing breast cancer and its microenvironment, by focusing on the biological and molecular mechanisms underlying the antineoplastic effects of these molecules.

### 2.3. Molecular Mechanisms of Selective Action of DHA and EPA Conjugates on Epithelial Breast Cancer Cells and Tumor Microenvironment.

The development of breast cancer, that remains the leading cause of cancer incidence and mortality in women worldwide [84], has been associated with genetic, hormonal, environmental, and nutritional factors. Among dietary factors, PUFAs have been implicated in breast cancer risk, although their role in the promotion or prevention of breast cancer development and progression in humans is not completely clarified and is still a matter of debate. Mounting evidence shows that dietary *n*−3 PUFAs and their derivatives may exert anti-carcinogenic actions mediated by growth-inhibition and apoptosis induction of breast cancer cells in vitro and in vivo, either alone or in combination with conventional anticancer therapies [25,27,30,31,34,85,86,87]. Importantly potential drug sensitizing effects of DHA and EPA have been reported [7,88]. In some studies, *n*−3 PUFAs were chemically conjugated with other drugs or formulated in nanoemulsion in an effort to provide new innovative therapeutic strategies for breast cancer [28,32,33,35]. Among multiple mode of actions, the anti-cancer activities exerted by EPA and DHA are also likely due to their ability to bind PPARγ [89]. It has been largely documented that induction of different pathways of apoptotic process is a biological response resulting from PPARγ activation in breast cancer cells [90,91,92,93]. In line with these findings, it has been shown that *n*-3 PUFAs exert anti-tumorigenic effects as direct agonists for PPARγ in breast cancer cells [73,94,95]. In addition, dietary supplementation with *n*-3 PUFAs was found to increase PPARγ protein content, concomitantly with a reduction of tumor burden in rats with induced mammary carcinogenesis [96]. Thus, PPARγ activation is beneficial for controlling breast cancer, suggesting a useful role for these molecules in the treatment of breast carcinoma.

#### 2.3.1. DHEA and EPEA as well as DHADA and EPADA

**Figure 3:**
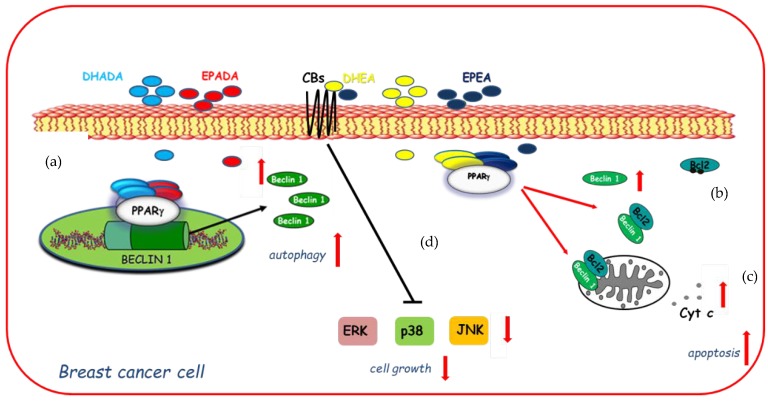
Molecular mechanisms by which docosahexaenoic acid (DHA)- and eicosapentaenoic acid (EPA)-conjugates upregulate Beclin-1 expression in a transcriptional-dependent (**a**) and -independent (**b**) manner through peroxisome proliferator activated receptor gamma (PPARγ) activation promoting autophagy in breast cancer cells and subsequently apoptosis (**c**) as evidenced by cytochrome *c* release (Cyt *c*) from mitochondria to the cytosol. In addition, DHEA and EPEA by binding to cannabinoid receptors (CBs) elicit changes in mitogen-activated protein kinase (MAPK) signaling (**d**) by decreasing activation of ERK (Extracellular Signal-regulated Kinase), p38 MAPK (Mitogen-Activated Protein Kinase), and JNK (c-Jun N-terminal kinase) MAPK pathways inducing cell growth inhibition.

On the basis of these findings and with the increasing focus towards the discovery of *n*−3 PUFA derived compounds with novel bioactivity, the ability of DHEA and EPEA as well as DHADA and EPADA to exert antineoplastic activities was investigated in breast cancer cell lines. Interestingly, Rovito and coworkers have demonstrated, for the first time, that both *N*-acylethanolamines and *N*-acyldopamines were able to exert inhibition of cancer cell proliferation, whereas they did not affect growth of nontumorigenic breast epithelial cells [22,23], appearing to be selective against cancer cells respect to normal cells. The molecular mechanism underlying this biological effect required the involvement of PPARγ activation. Specifically, both couples of *n*-3 PUFA conjugates were PPARγ-inducers able to activate the endogenous PPARγ and to upregulate its expression triggering autophagy in breast cancer cells [22,23]. The autophagy process is triggered by the activation of the key autophagy-related protein, Beclin-1 [97], whose function is also regulated by interaction with the anti-apoptotic protein Bcl-2 (B-cell lymphoma-2). Indeed, the binding of Beclin-1 with Bcl-2 homology-3 (BH3) domain results in the inhibition of Beclin-1 autophagic function [98], indicating Beclin-1 as a key molecule at the crossroad between autophagic and/or apoptotic cell death. The interaction between Beclin-1 and Bcl-2 might be reduced by phosphorylation of Bcl-2 by c-Jun N-terminal kinase 1 (JNK1) at residues threonine 69, serine 70, and serine 87 [99] thus promoting autophagy. In human breast cancer cells, DHEA and EPEA induced Bcl-2 phosphorylation at serine 70 reducing its physical association with Beclin-1 and leading to increased levels of Beclin-1 unbound protein, thus resulting in autophagosome formation [22]. Apart from the upregulation exerted by DHA and EPA conjugates with ethanolamine on Beclin-1 at non transcriptional levels, dopamine conjugates were also demonstrated to be able to induce a transcriptional regulation of Beclin-1. Of interest, DHADA and EPADA administration increased Beclin-1 levels by regulating its gene promoter transcriptional activity through direct recruitment of PPARγ, demonstrating a novel mechanism by which these molecules regulate autophagy [23]. A hypothetical model of molecular mechanisms by which DHA and EPA conjugates through PPARγ activation upregulate Beclin-1 expression in a transcriptional-dependent and -independent manner promoting autophagy in breast cancer cells is reported in Figure 3. These findings support the concept that autophagy plays a crucial role in the first phase of the antiproliferative action of these compounds. After long term treatment with DHADA and EPADA in breast cancer cells, an enhanced levels of Beclin-1 cleavage associated with activation of the apoptotic cascade (cytochrome *c* release from mitochondria to the cytosol) was found, highlighting the complex dialogue between autophagy and apoptosis induced by DHADA and EPADA in breast cancer cells [23]. Recently, Brown and coworkers have added novel findings regarding the anticancer effects of this class of signaling molecules demonstrating that *n*-3 PUFA ethanolamides exert antiproliferative properties against different breast cancer cells irrespective from their receptor status particularly reducing the migratory and invasive potential of more aggressive breast cancer cell lines [36]. These effects were mediated, at least in part, by binding and activation of CB receptors leading to reduced activation of p38, ERK and JNK mitogen-activated protein kinase (MAPK) pathways [36]. In line with previous findings [22], DHEA and EPEA did not influence cell growth of epithelial non-malignant breast cancer cells [36], highlighting that both compounds could be of interest in the pharmacological and nutritional research fields. The specific mechanisms involved in *n*-3 PUFA amide antineoplastic effects are not fully understood, but nowadays the involvement of PPARγ and CB receptors in mediating the growth inhibition, autophagy, and apoptosis exerted by these molecules in breast cancer cells is summarized in Figure 3.

#### 2.3.2. DHEA and DHA-5HT

Along with the anti-inflammatory and antineoplastic properties found for conjugates of the *n*−3 PUFAs [53], the effects of two DHA metabolites, DHEA and DHA-5HT on the polarization of human macrophages co-cultured with different breast cancer cells, that mimic the features of tumor associated macrophages within the tumor microenvironment have also been reported [100]. Interestingly, DHEA as well as DHA-5HT attenuated cytokine secretion by macrophages associated to breast cancer in a PPARγ dependent-manner [100]. Given the key role played by endogenous and synthetic PUFAs as PPARγ ligands in the crosstalk between cancer cells and tumor-associated macrophages, these mediators may represent novel tools in the therapeutic strategies that target both epithelial neoplastic cells and tumor microenvironment components.

**Figure 4:**
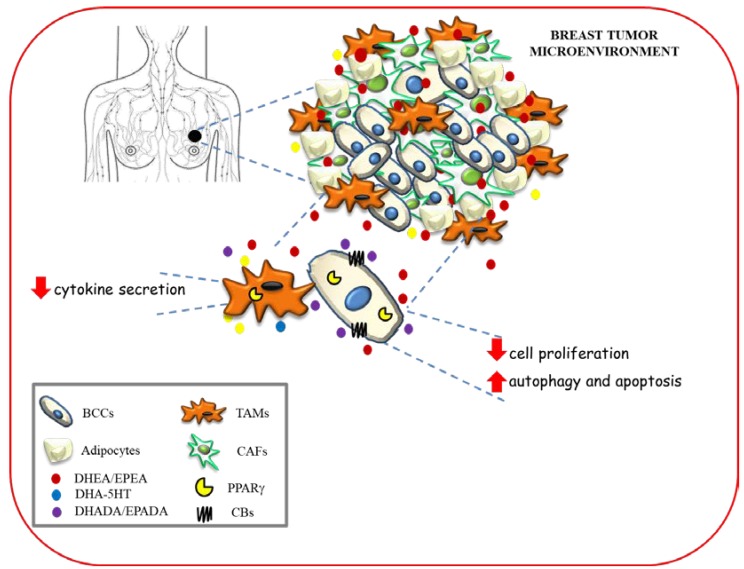
Hypothetical scheme showing multiple modes of action of *n*−3 PUFA amides in modulating breast cancer development and progression within tumor microenvironment. BCC: breast cancer cells; TAMs: tumor associated macrophages; CAFs: cancer associated fibroblasts.

The hypothetical scheme showing multiple modes of action of *n*−3 PUFA amides in modulating breast cancer development and progression within tumor microenvironment is depicted in Figure 4.

## 3. Conclusions

Breast cancer is the most challenging disease among all types of cancer, being the leading cause of cancer-related mortality in women worldwide. Despite hormonal therapy, chemotherapy and radiation after surgery represent first line treatments for breast cancer, there is a rising problem that patients can develop severe side effects and therapeutic resistance. Thus, many studies are focusing on natural nontoxic and dietary agents acting on multiple targets in an effort to provide a promising and cost-effective approach to reduce breast cancer incidence, morbidity, and mortality. In this context, in the past few decades, a significant amount of research has been carried out on the anticancer activities of *n*−3 PUFAs and their conjugates. These compounds have attracted much attention because of their potential roles in several pathophysiological conditions, suggesting that they could represent a new additional class of endogenous signaling molecules. Some of these *n*–3 PUFA amides exert immune-modulating effects and inhibition of breast cancer growth in in vitro and in vivo models acting as modulators of different cellular signaling pathways. Most importantly, the cytotoxic activity exerted by *n*-3 PUFAs and their derivatives appears to be selective against cancer cells without harming normal cells, whereas conventional chemotherapeutics kill malignant cells but in combination with other drugs have the potential to increase the sensitivity of tumor cells to conventional cytotoxic therapies, especially in more aggressive phenotypes that are resistant to treatments. Finally, pharmaceutical nanotechnologies can be applied to the formulation of lipid-based anticancer drugs designed to provide new innovative therapeutic strategies. Overall, the above considerations greatly encourage further in vitro research in order to fully comprehend the molecular mechanism of action of *n*-3 PUFA derivatives, the interplay with different biochemical routes and signaling pathways in breast cancer. Since conjugates of EPA and DHA possess several interesting biological properties, preclinical and clinical studies should be conducted to assess the potential of such compounds from a pharmacological or nutritional perspective as antineoplastic agents.

## Figures and Tables

**Table 1:** “In vitro” and “in vivo” studies showing the effects and the suggested mechanisms of polyunsaturated fatty acid derivatives in breast cancer.

Cell Lines *Animal Model*	Compounds	Mechanisms	Findings	Ref.
MCF-7	*N*-acyl dopamines	CB_1_ receptor	Anti-proliferative effects	[24]
MCF-7/ADR doxorubicin-resistant	EPADI	P-Glycoprotein	Sensitize cytotoxic effects of doxorubicin	[25]
MDA-MB-231	Propofol-DHA propofol-EPA	Unknown	Cell migration inhibition, apoptosis	[26]
MCF-7/Topo	TQ-Fatty Acid Conjugates	Unknown	Anti-proliferative effects	[27]
MDA-MB-231 MCF-7 AU565 MDA-MB-361	PP-DHA DIPP-DHA IPP-DHA CHP–DHA P–DHA	HDAC activity	Growth inhibition	[28]
MCF-7 MDA-MB-231	DHEA EPEA	Endogenous enzymes	Synthesis from parental DHA and EPA	[29]
MCF-7 MDA-MB-231	DOX–LNA	Endocytic transport	Improvement of DOX-LNA uptake and cytotoxicity	[30]
*MDA-MB-231 cell xenografts SCID mice*	DOX–LNA	Endocytic transport	Tumor growth inhibition	[31]
MCF-7	DHEA EPEA	PPARγ signaling	Growth inhibition, autophagy	[22]
MDA-MB-231 MDA-MB-468	LOV–DHA	Unknown	Growth inhibition, apoptosis	[32]
MCF-7 *MCF-7 bearing-nude mice*	DHA-GEM	Endocytic transport	Cytotoxicity, tumor growth inhibition	[33]
MCF-7 SKBR3 MDA-MB-231	DHADA EPADA	PPARγ signaling	Autophagy, apoptosis	[23]
MCF-7 SKBR-3 T47D MCF-10F trMCF bsMCF MDA-MB-231 BT-549	4-OH-DHA, 4-OXO-DHA	Unknown	Anti-proliferative effects	[34]
*MDA-MB-231 cell xenografts Balb/c nu/nu mice*	ClFPh-CHA-loaded nano-emulsion	Bioavailability	Tumor growth inhibition	[35]
MCF-7 MDA-MB-231	DHEA EPEA	CB receptors, p38-MAPK, JNK, ERK signaling pathways	Anti-proliferative effects, migration, and invasion inhibition	[36]

CHP–DHA: 2-cyclohexanephenol-docosahexaenoate; ClFPh-CHA: *n*−3 17,18-epoxyeicosanoic acid analogue; CB: cannabinoid receptor; DHA: docosahexaenoic acid; DHADA: DHA-dopamine (DA); DHA-GEM: gemcitabine-DHA; DHEA: docosahexaenoylethanolamine; DIPP–DHA: 2,4-diisopropylphenol-docosahexaenoate; DOX–LNA: doxorubicin-α-linolenic acid; EPA: eicosapentaenoic acid; EPADA: EPA-dopamine (DA); EPEA: eicosapentaenoylethanolamine; EPADI; eicosapentaenoic acid diester; FAAH: fatty acid amide hydrolase; IPP–DHA: 2-isopropylphenol–docosahexaenoate; LOV–DHA: lovastatin–docosahexaenoate; PE: phosphatidyl-ethanolamine; P–DHA: phenol–docosahexaenoate; PP–DHA: 2,6-diisopropylphenol–docosahexaenoate; PPARγ: peroxisome proliferator-activated receptor gamma; TQ: thymoquinone.

## Abbreviations

AA	arachidonic acid
ALA	alpha linolenic acid
Bcl-2	B-cell lymphoma-2
BH3	Bcl-2 homology-3
CALB	Candida antarctica lipase B
CB1	cannabinoid receptor 1
CB2	cannabinoid receptor 2
COX	cyclooxygenase
COX-2	cyclooxygenase-2
DHA	docosahexaenoic acid
2-DHG	docosahexaenoyl-glycerol
DHEA	docosahexaenoyl ethanolamine
DHADA	docosahexaenoyl dopamine
DHA-5HT	docosahexaenoyl serotonin
EPA	eicosapentaenoic acid
EPADA	eicosapentaenoyl dopamine
EPEA	eicosapentaenoyl ethanolamine
ERK	extracellular signal-regulated kinase
FAAH	fatty amide hydrolase
GPRs	G coupled protein receptors
IL-1β	interleukin-1beta
IL-6	interleukin-6
IL-17	interleukin-17
IL-23	interleukin-23
JNK1	c-Jun N-terminal kinase 1
LA	linoleic acid
LOX	lipoxygenase
LPS	lipopolysaccharide
MAPK	mitogen-activated protein kinase
MCP-1	monocyte chemoattractant protein-1
MIP3A	macrophage-inflammatory protein-3α
NO	nitric oxide
PGE2	prostaglandin E2
PPARγ	peroxisome proliferator activated receptor gamma
PPARs	peroxisome proliferator activated receptors
PRISMA	Preferred Reporting Items for Systematic Reviews and Meta-Analyses
PUFA	polyunsaturated fatty acids
Th17	T helper 17
TICs	tumor-initiating cells
TRVP1	transient receptors potential channel type V1

## References

[1] Anderson C., Milne G.L., Sandler D.P., Nichols H.B. (2016). Oxidative stress in relation to diet and physical activity among premenopausal women. Br. J. Nutr..

[2] Pauwels E.K., Kairemo K. (2008). Fatty acid facts, part II: Role in the prevention of carcinogenesis, or, more fish on the dish?. Drug News Perspect..

[3] Jolly C.A. (2005). Diet manipulation and prevention of aging, cancer and autoimmune disease. Curr. Opin. Clin. Nutr. Metab. Care.

[4] Wendel M., Heller A.R. (2009). Anticancer actions of omega-3 fatty acids--current state and future perspectives. Anticancer Agents Med. Chem..

[5] Martinez N., Herrera M., Frias L., Provencio M., Perez-Carrion R., Diaz V., Morse M., Crespo M.C. (2019). A combination of hydroxytyrosol, omega-3 fatty acids and curcumin improves pain and inflammation among early stage breast cancer patients receiving adjuvant hormonal therapy: Results of a pilot study. Clin. Transl. Oncol..

[6] Bjorklund G. (2015). The Adjuvant Nutritional Intervention in Cancer (ANICA) Trial. Nutr. Cancer.

[7] Siddiqui R.A., Harvey K.A., Xu Z., Bammerlin E.M., Walker C., Altenburg J.D. (2011). Docosahexaenoic acid: A natural powerful adjuvant that improves efficacy for anticancer treatment with no adverse effects. Biofactors.

[8] Blanckaert V., Ulmann L., Mimouni V., Antol J., Brancquart L., Chenais B. (2010). Docosahexaenoic acid intake decreases proliferation, increases apoptosis and decreases the invasive potential of the human breast carcinoma cell line MDA-MB-231. Int. J. Oncol..

[9] Chen H.W., Chao C.Y., Lin L.L., Lu C.Y., Liu K.L., Lii C.K., Li C.C. (2013). Inhibition of matrix metalloproteinase-9 expression by docosahexaenoic acid mediated by heme oxygenase 1 in 12-O-tetradecanoylphorbol-13-acetate-induced MCF-7 human breast cancer cells. Arch. Toxicol..

[10] Spencer L., Mann C., Metcalfe M., Webb M., Pollard C., Spencer D., Berry D., Steward W., Dennison A. (2009). The effect of omega-3 FAs on tumour angiogenesis and their therapeutic potential. Eur. J. Cancer.

[11] Derosa G., Cicero A.F., Fogari E., D’Angelo A., Bonaventura A., Romano D., Maffioli P. (2012). Effects of n-3 PUFAs on postprandial variation of metalloproteinases, and inflammatory and insulin resistance parameters in dyslipidemic patients: Evaluation with euglycemic clamp and oral fat load. J. Clin. Lipidol..

[12] Ravacci G.R., Brentani M.M., Tortelli T., Jr., Torrinhas R.S., Saldanha T., Torres E.A., Waitzberg D.L. (2013). Lipid raft disruption by docosahexaenoic acid induces apoptosis in transformed human mammary luminal epithelial cells harboring HER-2 overexpression. J. Nutr. Biochem..

[13] Ravacci G.R., Brentani M.M., Tortelli T.C., Torrinhas R.S., Santos J.R., Logullo A.F., Waitzberg D.L. (2015). Docosahexaenoic Acid Modulates a HER2-Associated Lipogenic Phenotype, Induces Apoptosis, and Increases Trastuzumab Action in HER2-Overexpressing Breast Carcinoma Cells. Biomed. Res. Int..

[14] Erickson K.L., Hubbard N.E. (2010). Fatty acids and breast cancer: The role of stem cells. Prostaglandins Leukot. Essent. Fatty Acids.

[15] Xiong A., Yu W., Liu Y., Sanders B.G., Kline K. (2016). Elimination of ALDH+ breast tumor initiating cells by docosahexanoic acid and/or gamma tocotrienol through SHP-1 inhibition of Stat3 signaling. Mol. Carcinog..

[16] Alexander S.P.H., Kendall D.A. (2007). The complications of promiscuity: Endocannabinoid action and metabolism. Br. J. Pharmacol..

[17] Di Marzo V., Bisogno T., De Petrocellis L. (2007). Endocannabinoids and related compounds: Walking back and forth between plant natural products and animal physiology. Chem. Biol..

[18] de Novellis V., Palazzo E., Rossi F., De Petrocellis L., Petrosino S., Guida F., Luongo L., Migliozzi A., Cristino L., Marabese I. (2008). The analgesic effect of N-arachidonoyl-serotonin, a FAAH inhibitor and TRPV1 receptor antagonist, associated with changes in rostral ventromedial medulla and locus coeruleus cell activity in rats. Neuropharmacology.

[19] Farrell E.K., Merkler D.J. (2008). Biosynthesis, degradation and pharmacological importance of the fatty acid amides. Drug Discov. Today.

[20] Connor M., Vaughan C.W., Vandenberg R.J. (2010). N-acyl amino acids and N-acyl neurotransmitter conjugates: Neuromodulators and probes for new drug targets. Br. J. Pharmacol..

[21] Ezzili C., Otrubova K., Boger D.L. (2010). Fatty acid amide signaling molecules. Bioorg. Med. Chem. Lett..

[22] Rovito D., Giordano C., Vizza D., Plastina P., Barone I., Casaburi I., Lanzino M., De Amicis F., Sisci D., Mauro L. (2013). Omega-3 PUFA ethanolamides DHEA and EPEA induce autophagy through PPARgamma activation in MCF-7 breast cancer cells. J. Cell Physiol..

[23] Rovito D., Giordano C., Plastina P., Barone I., De Amicis F., Mauro L., Rizza P., Lanzino M., Catalano S., Bonofiglio D. (2015). Omega-3 DHA- and EPA-dopamine conjugates induce PPARgamma-dependent breast cancer cell death through autophagy and apoptosis. Biochim. Biophys. Acta.

[24] Bisogno T., Melck D., Bobrov M., Gretskaya N.M., Bezuglov V.V., De Petrocellis L., Di Marzo V. (2000). N-acyl-dopamines: Novel synthetic CB(1) cannabinoid-receptor ligands and inhibitors of anandamide inactivation with cannabimimetic activity in vitro and in vivo. Biochem. J..

[25] Abulrob A.N., Mason M., Bryce R., Gumbleton M. (2000). The effect of fatty acids and analogues upon intracellular levels of doxorubicin in cells displaying P-glycoprotein mediated multidrug resistance. J. Drug Target..

[26] Siddiqui R.A., Zerouga M., Wu M., Castillo A., Harvey K., Zaloga G.P., Stillwell W. (2005). Anticancer properties of propofol-docosahexaenoate and propofol-eicosapentaenoate on breast cancer cells. Breast Cancer Res..

[27] Breyer S., Effenberger K., Schobert R. (2009). Effects of thymoquinone-fatty acid conjugates on cancer cells. ChemMedChem.

[28] Harvey K.A., Xu Z., Whitley P., Davisson V.J., Siddiqui R.A. (2010). Characterization of anticancer properties of 2,6-diisopropylphenol-docosahexaenoate and analogues in breast cancer cells. Bioorg. Med. Chem..

[29] Brown I., Wahle K.W., Cascio M.G., Smoum-Jaouni R., Mechoulam R., Pertwee R.G., Heys S.D. (2011). Omega-3 N-acylethanolamines are endogenously synthesised from omega-3 fatty acids in different human prostate and breast cancer cell lines. Prostaglandins Leukot. Essent. Fatty Acids.

[30] Huan M.L., Zhou S.Y., Teng Z.H., Zhang B.L., Liu X.Y., Wang J.P., Mei Q.B. (2009). Conjugation with alpha-linolenic acid improves cancer cell uptake and cytotoxicity of doxorubicin. Bioorg. Med. Chem. Lett..

[31] Huan M., Cui H., Teng Z., Zhang B., Wang J., Liu X., Xia H., Zhou S., Mei Q. (2012). In vivo anti-tumor activity of a new doxorubicin conjugate via alpha-linolenic acid. Biosci. Biotechnol. Biochem..

[32] Siddiqui R.A., Harvey K.A., Xu Z., Natarajan S.K., Davisson V.J. (2014). Characterization of lovastatin-docosahexaenoate anticancer properties against breast cancer cells. Bioorg. Med. Chem..

[33] Li S., Qin J., Tian C., Cao J., Fida G., Wang Z., Chen H., Qian Z., Chen W.R., Gu Y. (2014). The targeting mechanism of DHA ligand and its conjugate with Gemcitabine for the enhanced tumor therapy. Oncotarget.

[34] Pogash T.J., El-Bayoumy K., Amin S., Gowda K., de Cicco R.L., Barton M., Su Y., Russo I.H., Himmelberger J.A., Slifker M. (2015). Oxidized derivative of docosahexaenoic acid preferentially inhibit cell proliferation in triple negative over luminal breast cancer cells. In Vitro Cell Dev. Biol. Anim..

[35] Garrastazu Pereira G., Rawling T., Pozzoli M., Pazderka C., Chen Y., Dunstan C.R., Murray M., Sonvico F. (2018). Nanoemulsion-Enabled Oral Delivery of Novel Anticancer omega-3 Fatty Acid Derivatives. Nanomaterials.

[36] Brown I., Lee J., Sneddon A.A., Cascio M.G., Pertwee R.G., Wahle K.W.J., Rotondo D., Heys S.D. (2019). Anticancer effects of n-3 EPA and DHA and their endocannabinoid derivatives on breast cancer cell growth and invasion. Prostaglandins Leukot. Essent. Fatty Acids.

[37] Meijerink J., Balvers M., Witkamp R. (2013). N-Acyl amines of docosahexaenoic acid and other n-3 polyunsatured fatty acids—From fishy endocannabinoids to potential leads. Br. J. Pharmacol..

[38] Artmann A., Petersen G., Hellgren L.I., Boberg J., Skonberg C., Nellemann C., Hansen S.H., Hansen H.S. (2008). Influence of dietary fatty acids on endocannabinoid and N-acylethanolamine levels in rat brain, liver and small intestine. Biochim. Biophys. Acta.

[39] Berger A., Crozier G., Bisogno T., Cavaliere P., Innis S., Di Marzo V. (2001). Anandamide and diet: Inclusion of dietary arachidonate and docosahexaenoate leads to increased brain levels of the corresponding N-acylethanolamines in piglets. Proc. Natl. Acad. Sci. USA.

[40] Balvers M.G., Verhoeckx K.C., Plastina P., Wortelboer H.M., Meijerink J., Witkamp R.F. (2010). Docosahexaenoic acid and eicosapentaenoic acid are converted by 3T3-L1 adipocytes to N-acyl ethanolamines with anti-inflammatory properties. Biochim. Biophys. Acta.

[41] Kim H.Y., Moon H.S., Cao D., Lee J., Kevala K., Jun S.B., Lovinger D.M., Akbar M., Huang B.X. (2011). N-Docosahexaenoylethanolamide promotes development of hippocampal neurons. Biochem. J..

[42] Kim H.Y., Spector A.A., Xiong Z.M. (2011). A synaptogenic amide N-docosahexaenoylethanolamide promotes hippocampal development. Prostaglandins Other Lipid Mediat..

[43] Burstein S.H., Zurier R.B. (2009). Cannabinoids, endocannabinoids, and related analogs in inflammation. AAPS J..

[44] Bisogno T. (2008). Endogenous cannabinoids: Structure and metabolism. J. Neuroendocrinol..

[45] Wei B.Q., Mikkelsen T.S., McKinney M.K., Lander E.S., Cravatt B.F. (2006). A second fatty acid amide hydrolase with variable distribution among placental mammals. J. Biol. Chem..

[46] Fischer R., Konkel A., Mehling H., Blossey K., Gapelyuk A., Wessel N., von Schacky C., Dechend R., Muller D.N., Rothe M. (2014). Dietary omega-3 fatty acids modulate the eicosanoid profile in man primarily via the CYP-epoxygenase pathway. J. Lipid Res..

[47] Nording M.L., Yang J., Georgi K., Hegedus Karbowski C., German J.B., Weiss R.H., Hogg R.J., Trygg J., Hammock B.D., Zivkovic A.M. (2013). Individual variation in lipidomic profiles of healthy subjects in response to omega-3 Fatty acids. PLoS ONE.

[48] Astarita G., McKenzie J.H., Wang B., Strassburg K., Doneanu A., Johnson J., Baker A., Hankemeier T., Murphy J., Vreeken R.J. (2014). A protective lipidomic biosignature associated with a balanced omega-6/omega-3 ratio in fat-1 transgenic mice. PLoS ONE.

[49] Zelasko S., Arnold W.R., Das A. (2015). Endocannabinoid metabolism by cytochrome P450 monooxygenases. Prostaglandins Other Lipid Mediat..

[50] de Bus I., Witkamp R., Zuilhof H., Albada B., Balvers M. (2019). The role of n-3 PUFA-derived fatty acid derivatives and their oxygenated metabolites in the modulation of inflammation. Prostaglandins Other Lipid Mediat..

[51] Plastina P., Meijerink J., Vincken J.P., Gruppen H., Witkamp R., Gabriele B. (2009). Selective Synthesis of Unsaturated N-Acylethanolamines by Lipase-Catalyzed N-Acylation of Ethanolamine with Unsaturated Fatty Acids. Lett. Org. Chem..

[52] Bezuglov V., Bobrov M., Gretskaya N., Gonchar A., Zinchenko G., Melck D., Bisogno T., Di Marzo V., Kuklev D., Rossi J.C. (2001). Synthesis and biological evaluation of novel amides of polyunsaturated fatty acids with dopamine. Bioorg. Med. Chem. Lett..

[53] Watson J.E., Kim J.S., Das A. (2019). Emerging class of omega-3 fatty acid endocannabinoids & their derivatives. Prostaglandins Other Lipid Mediat..

[54] Pandey R., Mousawy K., Nagarkatti M., Nagarkatti P. (2009). Endocannabinoids and immune regulation. Pharmacol. Res..

[55] Cione E., Plastina P., Pingitore A., Perri M., Caroleo M.C., Fazio A., Witkamp R., Meijerink J. (2019). Capsaicin Analogues Derived from n-3 Polyunsaturated Fatty Acids (PUFAs) Reduce Inflammatory Activity of Macrophages and Stimulate Insulin Secretion by beta-Cells In Vitro. Nutrients.

[56] Meijerink J., Plastina P., Vincken J.P., Poland M., Attya M., Balvers M., Gruppen H., Gabriele B., Witkamp R.F. (2011). The ethanolamide metabolite of DHA, docosahexaenoylethanolamine, shows immunomodulating effects in mouse peritoneal and RAW264.7 macrophages: Evidence for a new link between fish oil and inflammation. Br. J. Nutr..

[57] Meijerink J., Poland M., Balvers M.G., Plastina P., Lute C., Dwarkasing J., van Norren K., Witkamp R.F. (2015). Inhibition of COX-2-mediated eicosanoid production plays a major role in the anti-inflammatory effects of the endocannabinoid N-docosahexaenoylethanolamine (DHEA) in macrophages. Br. J. Pharmacol..

[58] Balvers M.G., Verhoeckx K.C., Bijlsma S., Rubingh C.M., Meijerink J., Wortelboer H.M., Witkamp R.F. (2012). Fish oil and inflammatory status alter the n-3 to n-6 balance of the endocannabinoid and oxylipin metabolomes in mouse plasma and tissues. Metabolomics.

[59] Bobrov M.Y., Lyzhin A.A., Andrianova E.L., Gretskaya N.M., Zinchenko G.N., Frumkina L.E., Khaspekov L.G., Bezuglov V.V. (2006). Antioxidant and neuroprotective properties of N-docosahexaenoyl dopamine. Bull. Exp. Biol. Med..

[60] Dang H.T., Kang G.J., Yoo E.S., Hong J., Choi J.S., Kim H.S., Chung H.Y., Jung J.H. (2011). Evaluation of endogenous fatty acid amides and their synthetic analogues as potential anti-inflammatory leads. Bioorg. Med. Chem..

[61] Wang Y., Plastina P., Vincken J.P., Jansen R., Balvers M., Ten Klooster J.P., Gruppen H., Witkamp R., Meijerink J. (2017). N-Docosahexaenoyl Dopamine, an Endocannabinoid-like Conjugate of Dopamine and the n-3 Fatty Acid Docosahexaenoic Acid, Attenuates Lipopolysaccharide-Induced Activation of Microglia and Macrophages via COX-2. ACS Chem. Neurosci..

[62] Augimeri G., Plastina P., Gionfriddo G., Rovito D., Giordano C., Fazio A., Barone I., Catalano S., Ando S., Bonofiglio D. (2019). N-Eicosapentaenoyl Dopamine, A Conjugate of Dopamine and Eicosapentaenoic Acid (EPA), Exerts Anti-inflammatory Properties in Mouse and Human Macrophages. Nutrients.

[63] Verhoeckx K.C., Voortman T., Balvers M.G., Hendriks H.F., H M.W., Witkamp R.F. (2011). Presence, formation and putative biological activities of N-acyl serotonins, a novel class of fatty-acid derived mediators, in the intestinal tract. Biochim. Biophys. Acta.

[64] Poland M., ten Klooster J.P., Wang Z., Pieters R., Boekschoten M., Witkamp R., Meijerink J. (2016). Docosahexaenoyl serotonin, an endogenously formed n-3 fatty acid-serotonin conjugate has anti-inflammatory properties by attenuating IL-23-IL-17 signaling in macrophages. Biochim. Biophys. Acta Mol. Cell Biol. Lipids.

[65] Wang Y., Balvers M.G.J., Hendriks H.F.J., Wilpshaar T., van Heek T., Witkamp R.F., Meijerink J. (2017). Docosahexaenoyl serotonin emerges as most potent inhibitor of IL-17 and CCL-20 released by blood mononuclear cells from a series of N-acyl serotonins identified in human intestinal tissue. Biochim. Biophys. Acta Mol. Cell Biol. Lipids.

[66] McDougle D.R., Watson J.E., Abdeen A.A., Adili R., Caputo M.P., Krapf J.E., Johnson R.W., Kilian K.A., Holinstat M., Das A. (2017). Anti-inflammatory omega-3 endocannabinoid epoxides. Proc. Natl. Acad. Sci. USA.

[67] Yang R., Fredman G., Krishnamoorthy S., Agrawal N., Irimia D., Piomelli D., Serhan C.N. (2011). Decoding functional metabolomics with docosahexaenoyl ethanolamide (DHEA) identifies novel bioactive signals. J. Biol. Chem..

[68] Alharthi N., Christensen P., Hourani W., Ortori C., Barrett D.A., Bennett A.J., Chapman V., Alexander S.P.H. (2018). n-3 polyunsaturated N-acylethanolamines are CB2 cannabinoid receptor-preferring endocannabinoids. Biochim. Biophys. Acta Mol. Cell Biol. Lipids.

[69] Desvergne B., Wahli W. (1999). Peroxisome proliferator-activated receptors: Nuclear control of metabolism. Endocr. Rev..

[70] Georgiadi A., Kersten S. (2012). Mechanisms of gene regulation by fatty acids. Adv. Nutr..

[71] Gurnell M., Savage D.B., Chatterjee V.K., O’Rahilly S. (2003). The metabolic syndrome: Peroxisome proliferator-activated receptor gamma and its therapeutic modulation. J. Clin. Endocrinol. Metab..

[72] Kliewer S.A., Sundseth S.S., Jones S.A., Brown P.J., Wisely G.B., Koble C.S., Devchand P., Wahli W., Willson T.M., Lenhard J.M. (1997). Fatty acids and eicosanoids regulate gene expression through direct interactions with peroxisome proliferator-activated receptors alpha and gamma. Proc. Natl. Acad. Sci. USA.

[73] Flachs P., Rossmeisl M., Bryhn M., Kopecky J. (2009). Cellular and molecular effects of n-3 polyunsaturated fatty acids on adipose tissue biology and metabolism. Clin. Sci..

[74] Chang H.Y., Lee H.N., Kim W., Surh Y.J. (2015). Docosahexaenoic acid induces M2 macrophage polarization through peroxisome proliferator-activated receptor gamma activation. Life Sci..

[75] Gutierrez S., Svahn S.L., Johansson M.E. (2019). Effects of Omega-3 Fatty Acids on Immune Cells. Int. J. Mol. Sci..

[76] Vaughan V.C., Hassing M.R., Lewandowski P.A. (2013). Marine polyunsaturated fatty acids and cancer therapy. Br. J. Cancer.

[77] Lin G., Zhu S., Wu Y., Song C., Wang W., Zhang Y., Chen Y.L., He Z. (2017). omega-3 free fatty acids and all-trans retinoic acid synergistically induce growth inhibition of three subtypes of breast cancer cell lines. Sci. Rep..

[78] Apaya M.K., Shiau J.Y., Liao G.S., Liang Y.J., Chen C.W., Yang H.C., Chu C.H., Yu J.C., Shyur L.F. (2019). Integrated omics-based pathway analyses uncover CYP epoxygenase-associated networks as theranostic targets for metastatic triple negative breast cancer. J. Exp. Clin. Cancer Res..

[79] Pizato N., Kiffer L., Luzete B.C., Assumpcao J.A.F., Correa L.H., Melo H.A.B., Sant’Ana L.P., Ito M.K., Magalhaes K.G. (2019). Omega 3-DHA and Delta-Tocotrienol Modulate Lipid Droplet Biogenesis and Lipophagy in Breast Cancer Cells: The Impact in Cancer Aggressiveness. Nutrients.

[80] Garcia C.P., Lamarque A.L., Comba A., Berra M.A., Silva R.A., Labuckas D.O., Das U.N., Eynard A.R., Pasqualini M.E. (2015). Synergistic anti-tumor effects of melatonin and PUFAs from walnuts in a murine mammary adenocarcinoma model. Nutrition.

[81] Xiong A., Yu W., Tiwary R., Sanders B.G., Kline K. (2012). Distinct roles of different forms of vitamin E in DHA-induced apoptosis in triple-negative breast cancer cells. Mol. Nutr. Food Res..

[82] Gaston R., Maria Eugenia P., Das U.N., Eynard A.R. (2017). Polyunsaturated Fatty Acids Differentially Modulate Cell Proliferation and Endocannabinoid System in Two Human Cancer Lines. Arch. Med. Res..

[83] Fabian C.J., Kimler B.F., Hursting S.D. (2015). Omega-3 fatty acids for breast cancer prevention and survivorship. Breast Cancer Res..

[84] Bray F., Ferlay J., Soerjomataram I., Siegel R.L., Torre L.A., Jemal A. (2018). Global cancer statistics 2018: GLOBOCAN estimates of incidence and mortality worldwide for 36 cancers in 185 countries. CA Cancer J. Clin..

[85] Corsetto P.A., Colombo I., Kopecka J., Rizzo A.M., Riganti C. (2017). Omega-3 Long Chain Polyunsaturated Fatty Acids as Sensitizing Agents and Multidrug Resistance Revertants in Cancer Therapy. Int. J. Mol. Sci..

[86] Rawling T., Choucair H., Koolaji N., Bourget K., Allison S.E., Chen Y.J., Dunstan C.R., Murray M. (2017). A Novel Arylurea Fatty Acid That Targets the Mitochondrion and Depletes Cardiolipin to Promote Killing of Breast Cancer Cells. J. Med. Chem..

[87] Bonofiglio D., Giordano C., De Amicis F., Lanzino M., Ando S. (2016). Natural Products as Promising Antitumoral Agents in Breast Cancer: Mechanisms of Action and Molecular Targets. Mini Rev. Med. Chem..

[88] Calviello G., Serini S., Piccioni E., Pessina G. (2009). Antineoplastic effects of n-3 polyunsaturated fatty acids in combination with drugs and radiotherapy: Preventive and therapeutic strategies. Nutr. Cancer.

[89] Gani O.A. (2008). Are fish oil omega-3 long-chain fatty acids and their derivatives peroxisome proliferator-activated receptor agonists?. Cardiovasc. Diabetol..

[90] Bonofiglio D., Aquila S., Catalano S., Gabriele S., Belmonte M., Middea E., Qi H., Morelli C., Gentile M., Maggiolini M. (2006). Peroxisome proliferator-activated receptor-gamma activates p53 gene promoter binding to the nuclear factor-kappaB sequence in human MCF7 breast cancer cells. Mol. Endocrinol..

[91] Bonofiglio D., Cione E., Qi H., Pingitore A., Perri M., Catalano S., Vizza D., Panno M.L., Genchi G., Fuqua S.A. (2009). Combined low doses of PPARgamma and RXR ligands trigger an intrinsic apoptotic pathway in human breast cancer cells. Am. J. Pathol..

[92] Bonofiglio D., Cione E., Vizza D., Perri M., Pingitore A., Qi H., Catalano S., Rovito D., Genchi G., Andò S. (2011). Bid as a potential target of apoptotic effects exerted by low doses of PPARgamma and RXR ligands in breast cancer cells. Cell Cycle.

[93] Bonofiglio D., Gabriele S., Aquila S., Qi H., Belmonte M., Catalano S., Andò S. (2009). Peroxisome proliferator-activated receptor gamma activates fas ligand gene promoter inducing apoptosis in human breast cancer cells. Breast Cancer Res. Treat..

[94] Clay C.E., Namen A.M., Atsumi G., Willingham M.C., High K.P., Kute T.E., Trimboli A.J., Fonteh A.N., Dawson P.A., Chilton F.H. (1999). Influence of J series prostaglandins on apoptosis and tumorigenesis of breast cancer cells. Carcinogenesis.

[95] Barascu A., Besson P., Le Floch O., Bougnoux P., Jourdan M.L. (2006). CDK1-cyclin B1 mediates the inhibition of proliferation induced by omega-3 fatty acids in MDA-MB-231 breast cancer cells. Int. J. Biochem. Cell Biol..

[96] Jiang W., Zhu Z., McGinley J.N., El Bayoumy K., Manni A., Thompson H.J. (2012). Identification of a molecular signature underlying inhibition of mammary carcinoma growth by dietary N-3 fatty acids. Cancer Res..

[97] Liang X.H., Jackson S., Seaman M., Brown K., Kempkes B., Hibshoosh H., Levine B. (1999). Induction of autophagy and inhibition of tumorigenesis by beclin 1. Nature.

[98] Pattingre S., Tassa A., Qu X., Garuti R., Liang X.H., Mizushima N., Packer M., Schneider M.D., Levine B. (2005). Bcl-2 antiapoptotic proteins inhibit Beclin 1-dependent autophagy. Cell.

[99] Wei Y.J., Pattingre S., Sinha S., Bassik M., Levine B. (2008). JNK1-mediated phosphorylation of BcI-2 regulates starvation-induced autophagy. Mol. Cell.

[100] Gionfriddo G., Plastina P., Augimeri G., Catalano S., Giordano C., Barone I., Morelli C., Giordano F., Gelsomino L., Sisci D. (2020). Modulating Tumor-Associated Macrophage Polarization by Synthetic and Natural PPARgamma Ligands as a Potential Target in Breast Cancer. Cells.

